# Antiviral foods in the battle against viral infections: Understanding the molecular mechanism

**DOI:** 10.1002/fsn3.3454

**Published:** 2023-05-22

**Authors:** Md. Shofiul Azam, Md. Nahidul Islam, Md. Wahiduzzaman, Mahabub Alam, Akib Atique Khan Dhrubo

**Affiliations:** ^1^ Department of Food Engineering Dhaka University of Engineering & Technology Gazipur Bangladesh; ^2^ Department of Agro‐Processing Bangabandhu Sheikh Mujibur Rahman Agricultural University Gazipur Bangladesh; ^3^ Institute of Food Safety and Processing Bangabandhu Sheikh Mujibur Rahman Agricultural University Gazipur Bangladesh; ^4^ Bio‐Med Big Data Center, CAS Key Laboratory of Computational Biology, CAS‐MPG Partner Institute for Computational Biology, Shanghai Institute of Nutrition and Health University of Chinese Academy of Sciences, Chinese Academy of Sciences Shanghai China; ^5^ Department of Food Engineering and Tea Technology Shahjalal University of Science and Technology Sylhet Bangladesh; ^6^ Department of Chemical Engineering Dhaka University of Engineering & Technology Gazipur Bangladesh

**Keywords:** antiviral food, COVID‐19, immunity, probiotics, viral infections

## Abstract

Viruses produce a variety of illnesses, which may also cause acute respiratory syndrome. All viral infections, including COVID‐19, are associated with the strength of the immune system. Till now, traditional medicine or vaccines for most viral diseases have not been effective. Antiviral and immune‐boosting diets may provide defense against viral diseases by lowering the risk of infection and assisting rapid recovery. The purpose of this review was to gather, analyze, and present data based on scientific evidence in order to provide an overview of the mechanistic insights of antiviral bioactive metabolites. We have covered a wide range of food with antiviral properties in this review, along with their potential mechanism of action against viral infections. Additionally, the opportunities and challenges of using antiviral food have been critically reviewed. Bioactive plant compounds, not only help in maintaining the body's normal physiological mechanism and good health but are also essential for improving the body's immunity and therefore can be effective against viral diseases. These agents fight viral diseases either by incorporating the body's defense mechanism or by enhancing the cell's immune system. Regular intake of antiviral foods may prevent future pandemic and consumption of these antiviral agents with traditional medicine may reduce the severity of viral diseases. Therefore, the synergistic effect of antiviral foods and medication needs to be investigated.

## Graphical abstract

Regular intake of antiviral foods may prevent future pandemic and the use of antiviral food with traditional medicine may reduce the severity of viral diseases.

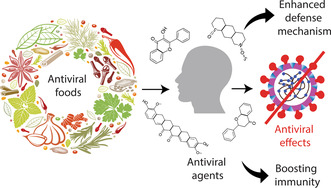

## 1 INTRODUCTION

Virus infections are still a leading source of illness and mortality in many parts of the world. The most hazardous viral infections include Ebola, Influenza, AIDS (acquired immunodeficiency syndrome), Dengue fever, and SARS (severe acute respiratory syndrome). Each year, for example, influenza causes roughly 3 million occurrences of severe illness and 300,000–500,000 deaths. Every year, the number of people diagnosed with viral infections rises rapidly due to point mutation, recombination, and reassortment mechanism of the viral genome, population growth, urbanization, the increasing ease of international travel of human and virus‐infected animals or arthropods, trans‐species transmission of the viral agent, monocultures of genetically identical individuals, drug resistance, deforestation, global warming, intensive farming practices, and degradation of the environment.

Our world nowadays is confronted with a slew of emerging infectious diseases including HIV infections, SARS, lyme disease, *Escherichia coli* O157:H7, hantavirus, dengue fever, west Nile virus, and the zika virus (Liu et al., 2020). Viruses capable of causing respiratory, gastrointestinal, and urogenital infections, both old and novel, are now posing a greater threat. The number of viruses resistant to first‐line treatment, that is, Intravenous acyclovir is the first‐line treatment for Herpes simplex virus (HSV) encephalitis medications is rapidly increasing, necessitating the development of innovative antiviral agents (Andersen et al., 2020). Several reports show SARS‐CoV‐2 resistance to the drug PAXLOVID during the recent COVID‐19 pandemic (Hu et al., 2022; Iketani et al., 2023).

Because the most prevalent viruses can transmit from person to person, people in developing and developed countries are susceptible to fatal infectious diseases that can spread quickly. Researchers throughout the world are finding it difficult to prevent or treat them due to various factors such as simple mutations, high dissemination capacity, resistant viral pathogens, the emergence of new viral strains, and antibiotic inefficacy (Smith et al., 2018).

Antiviral agents are not actually virus‐containing vaccinations nor any antibodies that have a clear demonstrable preventive or curative impact on the virus‐infected host (Ruwali et al., 2013). Antiviral substances aid the body's defense against hazardous viruses. The hunt for efficient and reasonably priced medicines to meet the demands of the present has recently shifted to include medicinal plants and their bioactive metabolites (Kuchta & Cameron, 2021). Indigenous traditional herbal therapy has a long history of treating a wide range of chronic and infectious disorders. As a result, the search for new antiviral medicines concentrates on both synthetic combinations and metabolites originating from plants. Numerous plant metabolites have the ability to prevent viral replication with little to no negative effects on human's physiology (Bhuiyan et al., 2020). These natural compounds may have the ability to alter the host's immune responses to viral infections in addition to directly interfering with viral replication.

By merging current knowledge and understanding of virus‐caused diseases, the study's objective was to critically analyze the most recent update on the disease's course. The major objectives were to compile, critically evaluate, and offer an overview of the mechanistic insights of antiviral bioactive metabolites together with scientific evidence‐based data collection, analysis, and presentation.

## 2 FOOD THAT DECREASES VIRUS LOAD

### 2.1 Fermented food

**FIGURE 1:**
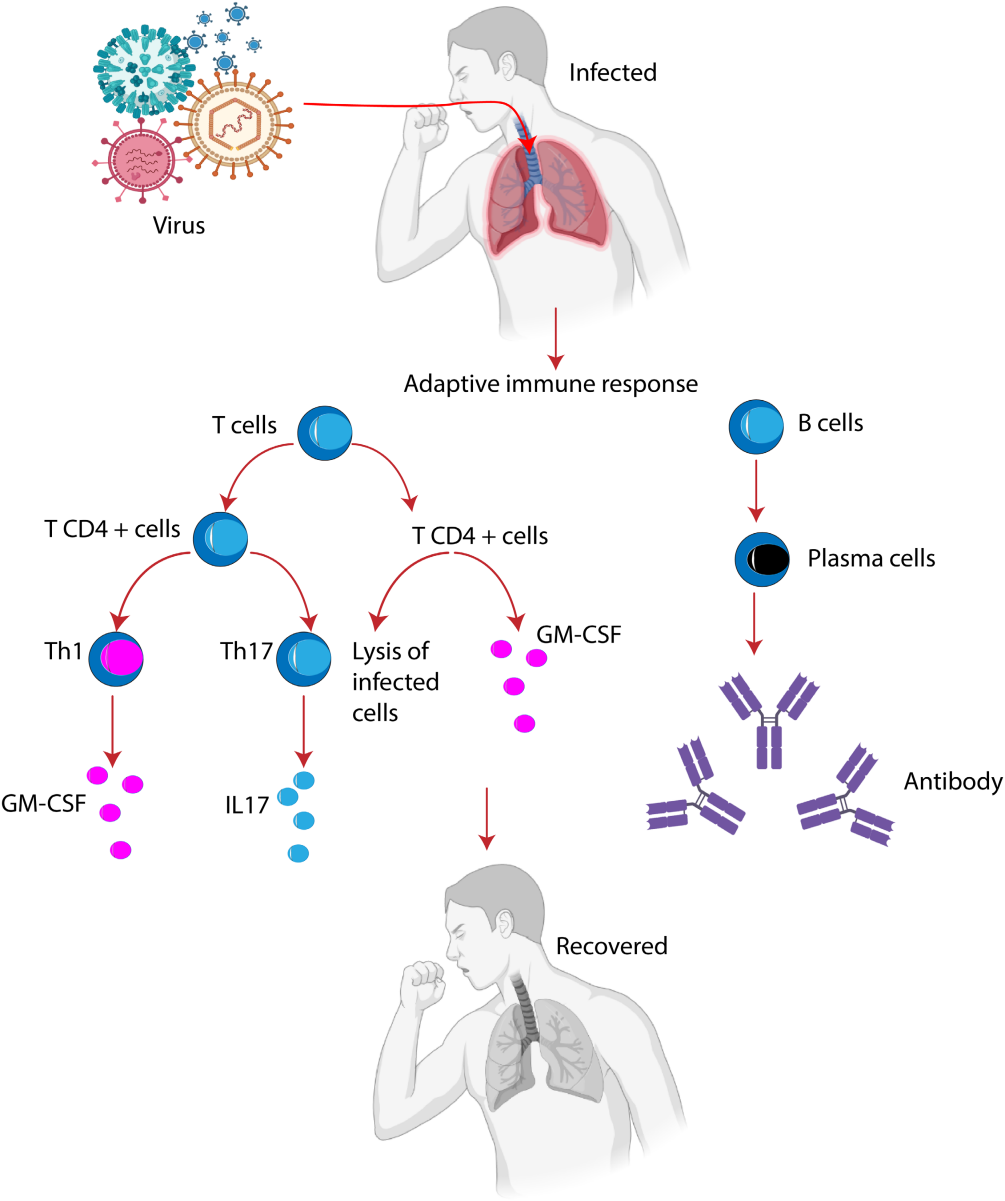
The adaptive immune response to viral pathogenesis.

Due to reports of significant antiviral activity, fermented foods and associated probiotic microorganisms have recently gotten a lot of attention. Several probiotic strains showed a diverse variety of antiviral properties and modes of action. The probiotic bacteria and bioactive ingredients in fermented products have antiviral capabilities that can combat viruses that affect the digestive and respiratory systems. Probiotics and bioactive compounds rich food could potentially hinder viruses from attaching to host cells and activate the immune system of the individual via increased natural killer cell toxicity, increased generation of pro‐inflammatory cytokines, and increased cytotoxicity of T cells (CD3+, CD16+, CD56+) (Muhialdin et al., 2021). Figure 1 represents the mechanism of the adaptive immune response to viral pathogenesis.

#### 2.1.1 Fermented vegetables

The antioxidant characteristics of many fermented vegetables make them nutritious, and they may help lower infection levels. Traditional fermented vegetable consumption for example is relatively high in some of the countries found to be low fatality from COVID‐19 (Fonseca et al., 2020).

**FIGURE 2:**
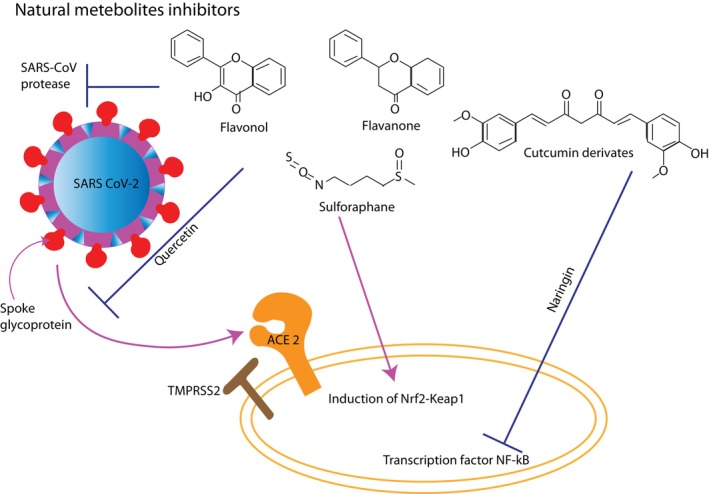
Potential COVID‐19 defense mechanisms in fermented vegetables. Adapted from Ghidoli et al. (2021).

In humans, (erythroid‐derived 2)‐like 2 (Nrf2) is the most powerful antioxidant and is able to inhibit the Angiotensin II Type I Receptor (AT1R) axis in particular (Bousquet et al., 2021). Sulforaphane, the most powerful natural activator of Nrf2, is found in cabbage as a precursor. *Lactobacilli*, which are similarly powerful Nrf2 activators, and are abundant in fermented vegetables (Bousquet et al., 2021). Therefore, fermented vegetables are a proof‐of‐concept for dietary changes that could increase the antioxidant level associated with Nrf2 and lessen the severity of COVID‐19 (Bousquet et al., 2021). Figure 2 illustrates the potential COVID‐19 defense mechanisms of fermented vegetables.

**FIGURE 3:**
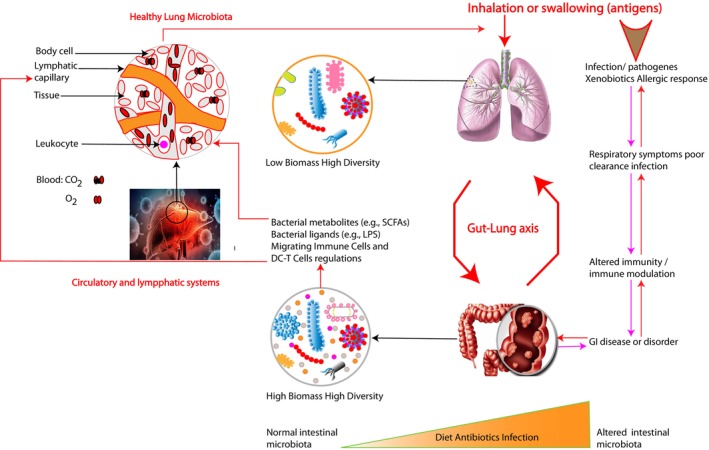
Possible link between diet, the gut‐lung axis, and host defense by the intestinal microbiota and lung immunity. Adapted from Samuelson et al. (2015).

Kimchi is a familiar conventional Korean fermented vegetable rich in minerals, vitamins, and other health‐promoting substances. The anti‐aging, ‐obesity, ‐mutagenic, ‐oxidant, ‐cancer, and ‐diabetic properties of kimchi are only a few of its health benefits. According to Choi et al. (2010), a variant of *Lactiplantibacillus plantarum* (YML009) obtained in Kimchi demonstrated remedial activity against the H1N1 influenza virus. Park et al. (2013) also discovered that intranasal and oral injection of *Lactiplantibacillus plantarum* (DK119) also collected from Kimchi increases mouse resistance against the H1N1 influenza virus. Furthermore, Kimchi's *Lactobacillus plantarum* Ln1 strains have recently been found to have immune‐stimulating properties (Jang et al., 2020). In the context of the SARS‐CoV‐2 virus, there is a link between a high intake of fermented cabbage and vegetables, which is popular in Central Europe, East Asia, and the Balkans, and decreased fatality rates (Bousquet et al., 2021). Figure 3 presents the possible link between diet, the gut‐lung axis, and host defense by the intestinal microbiota and lung immunity.

#### 2.1.2 Fermented milk

Yogurt is a very effective immune‐modulating meal with potential therapeutic effects due to its ability to alter the microbiome of the gastrointestinal tract (Kok & Hutkins, 2018). The growth of harmful bacteria is suppressed as the number of beneficiary bacteria grows, which helps to minimize infection (Meydani & Ha, 2000). Yogurt consumption has been shown in human studies to increase cytokine synthesis, antibody formation, and various cell activities, all of which help fight illnesses. Studies showed that a higher intake of low‐fat yogurt had a protective role on COVID‐19 (Darand et al., 2022). According to Choi et al. (2009), probiotic yogurt has a powerful anti‐simian rotavirus effect, neutralizing the virus and its toxins. Antiviral properties in lactic acid bacteria‐fermented yogurt may be effective against Enterovirus 71 (EV71) (Choi et al., 2010) and RNA viruses (Choi et al., 2009).

Diarrhoeal disorder in newborns and young kids is initiated by rotaviruses. *Lactobacillus* species (*L. casei* and *L. acidophilus*) and *Bifidobacterium* species (*B. longum*) have been demonstrated to have anti‐rotavirus effects in several investigations (Cantú‐Bernal et al., 2020). NSP4 protein generation and release of Ca^2+^, as well as a reduction in cell death, are the main mechanism to demonstrate the antiviral mechanism (Hamida et al., 2021).

Kefir is a milk‐based beverage prepared from kefir grains that resembles a less thick yogurt. It may improve the defense mechanism to fight viral diseases. Macrophage generation, increasing phagocytic activity, enhancing the yield of cluster of differentiation‐positive (CD4+), CD8+, immunoglobulin (Ig)G+ and IgA+ B cells, T cells, neutrophils, as well as cytokines (e.g., interleukin (IL)‐2, IL‐12, and interferon gamma‐γ) are some of the mechanisms of action of kefir (Hamida et al., 2021). Kefir can reduce IL‐6, IL‐1, TNF‐, and interferon expression, which has anti‐inflammatory effects (Vieira et al., 2021). Kefir may therefore be a potent inhibitor of the “cytokine storm” that fuels COVID‐19 (Hamida et al., 2021).

Lactoferrin (LF) is a glycoprotein that naturally occurs in various mucosal secretions. It has the ability to bind with iron, is non‐toxic, and has been found to possess immunomodulatory and anti‐inflammatory properties. LF is crucial in the initial line of defense against microbial infections (Siqueiros‐Cendón et al., 2014; Vorland, 1999). LF's antiviral properties are not limited to specific viruses and can combat a broad range of animal and human viruses, including both RNA and DNA viruses (Kell et al., 2020; Wang et al., 2020). Researchers have reported that LF exhibits a wide‐ranging ability to combat common human coronaviruses, and they have also explored its mechanism of action. The antiviral mechanism of action of bovine LF is through binding to host cell surface heparan sulfate proteoglycans (HSPGs) (Hu et al., 2021).

### 2.2 Herbs

Antiviral herbs such as oregano, tulsi, fennel, peppermint, pokeweed, black nightshade, and aloe vera boost defense and may be used to treat respiratory diseases such as mucus congestion, which can be a breeding ground for germs and viruses. Consuming tea made from ginger, garlic, and a tablespoon of thyme and oregano is associated with reduced odds of respiratory diseases (Goyal et al., 2021). The mechanism of action of many herbs has been proved by clinical trials. For example, in a recent clinical trial, *Polypodium leucotomos*, a tropical fern extract prevented the infection processes in athletes by enhancing their immune system. Besides, in vitro, studies on polypody extract have demonstrated its pleiotropic effect on different cytokines of the immune system (Sánchez‐Rodríguez et al., 2018).

#### 2.2.1 Oregano

Oregano (*Origanum vulgare*) plant is well‐known for its medicinal properties. Antiviral qualities are conferred by the presence of the active plant component carvacrol, which protects against viral infection. Oregano oil has protective activity against several viruses such as the HSV type 1, rotavirus, and respiratory virus, according to several researches (Sharifi‐Rad et al., 2017). Several in vitro and in vivo studies have shown multiple pharmacological properties such as anticancer, anti‐fungal, anti‐bacterial, anti‐oxidant, anti‐inflammatory, vasorelaxant, hepatoprotective, and spasmolytic (Javed et al., 2021). Daily use of oregano may help to stimulate the antiviral response (Hamidpour et al., 2014; Pilau et al., 2011). Carvacrol, received special attention due to recent reports of its specific binding with M^pro^, a protease enzyme in the viral genome belonging to non‐structural proteins showing a significant effect in the replication and maturation of SARS‐CoV‐2 (Kumar et al., 2021). In another recent study, carvacrol, a bioactive molecule in the EO of Ammoides verticillata Briq. was reported to inhibit ACE2 activity and suggested that it may block the host cell entry of SARS‐CoV‐2 (Abdelli et al., 2021).

#### 2.2.2 Tulsi

The main medical benefits of Tulsi (*Ocimum tenuiflorum*) are its powerful antiviral, anti‐inflammatory, antioxidant, and antibacterial capabilities (Goel, 2013). Apigenin and ursolic acid, two of the substances found in Tulsi extracts, have been shown to be fighting against several viruses such as herpes, hepatitis B, and enterovirus. Consuming Tulsi tea can improve the immune system and fight all types of cold, and respiratory inflammations which has become a common practice in South Asia (Kumar et al., 2013). Jain et al. (2022) used a molecular docking system to assess the potential of Tulsi as an adjunct therapy for the prevention and symptomatic treatment of COVID‐19. Authors reported that among vicenin, caryophyllene, cirsimaritin, isothymusin, and isothymonin, vicenin exhibited better binding energy, that is, −7.02 kcal/mol to COVID‐19 main protease through some responsible interactions to inhibit the replication of SARS‐CoV‐2 in the human body. In addition, tulsi has the potential to boost the immunity (Ugemuge & Paunikar, 2022).

#### 2.2.3 Fennel

Fennel (*Foeniculum vulgare*) is a licorice‐flavored herb that triggers immunity, reduces body's inflammation, and fights viral infection. Trans‐anethole, one of the active compounds in fennel seeds, is known for fighting against herpes viruses (Ibrahim & Moussa, 2021). Apart from this, the presence of strong antioxidants like vitamins A and C boosts immune activity. Phlegm, sinus, and respiratory inflammations can be treated with fennel tea or fennel‐derived drinks (Yakut et al., 2020). In a recent study, the antiviral activities of fennel extract, showed moderate antiviral activities 21.95% and 13.14% against the HSV and Coxsackievirus B4 viruses respectively (Suleiman & Helal, 2022).

#### 2.2.4 Peppermint

The essential oils of peppermint (*Mentha × piperita*) leaves are mainly composed of menthol, menthone, neomenthol, and iso‐menthone, with strong anti‐inflammatory, antibacterial, antiviral, scolicidal, immunomodulatory, antitumor, neuroprotective, antifatigue, and antioxidant activities (Saleh et al., 2021; Zhao et al., 2022). Peppermint oil's antiviral activity was shown to be strong against HSV‐1, and HSV‐2 (Civitelli et al., 2014; Nolkemper et al., 2006). Peppermint oil was applied to viruses at different periods of infection to discover the mode of antiviral action. When the HSV was pretreated with peppermint oil before adsorption, both herpes viruses were strongly suppressed (Schuhmacher et al., 2003). Peppermint extract showed strong antiviral activity against is human respiratory syncytial virus (HRSV), a syncytial virus that causes respiratory infection (Li et al., 2017). A recent study shows that peppermint helped to prevent the interaction between SARS‐CoV‐2 and the host body by inhibiting the formation of a receptor complex of viral spike protein and ACE2 receptors of the host body (Chakraborty et al., 2022).

#### 2.2.5 Black nightshade

Black nightshade (*Solanum nigrum*), is a weed that can be used as a tonic, anti‐inflammatory, antioxidant, and diuretic in the medication of a variety of disorders, including pneumonia, sore teeth, stomach discomfort, tonsillitis, wing worms, pain, inflammation, fever, and tumors (Taran et al., 2022). The herb has a long history of widespread utilization as a traditional cure for some life‐threatening diseases including tuberculosis, ulcer liver disorders, and even in cancer treatment (Kuete, 2014). Black nightshade extracts have been tested for their ability to suppress HIV‐1 (Javed et al., 2011).

#### 2.2.6 Aloe vera

Aloe vera (*Aloe barbadensis miller*) plant has antiviral activity against a variety of viruses, including the coronavirus SARS‐CoV‐1, HSV type 1, HSV type 2, Hemorrhagic Viral Rhobdavirus Septicaemia (VHS), HIV, Varicella‐Zoster virus, Influenza virus, Cytomegalovirus, poliovirus, and Human papillomavirus (Kilembe et al., 2020). Compounds from this plant have been demonstrated to be effective against the above‐mentioned viruses through processes such as virus enzyme interaction, viral envelope destruction, and so on (Mandadi & Scholthof, 2013). Also, the presence of high levels of zinc, which have been shown to diminish the effects of SARS‐CoV‐1, supports aloe vera's antiviral activity (Bongo et al., 2020).

#### 2.2.7 Tea

Tea (*Camellia sinensis*) is one of the polyphenolic substances whose antiviral activity is based on their antioxidant properties, as well as their ability to inhibit enzymes, break cell membranes, block viral binding and penetration into cells, and activate the host cell's self‐defense systems (Yiannakopoulou, 2012). Observational studies have reported the association between tea consumption and the risk of lowering respiratory tract infections (LRTIs) caused by viruses (HRSV, influenza virus, human coronavirus [hCoV], etc.) (Chen et al., 2023).

In a study conducted by Friedman (2007), the antiviral activity of a combination of four theaflavins isolated from black tea was much stronger (synergistic) than the sum of the activities of the four separate components. Steric and conformational influences appear to regulate viral infectivity, according to molecular modeling of the structures of the four theaflavins.

#### 2.2.8 Coffee

Naturally caffeinated drinks have long been recognized to provide a variety of health advantages and disease prevention. Simon et al. (2022) found in an epidemiological study that drinking two to three cups of coffee per day is linked to a lower risk of metabolic illnesses, which are frequently associated with a weakened immune system. Caffeic acid has also been found to have an antiviral effect against the DNA virus. (HSV) and the RNA virus (Polio virus), suppressing virus‐infected cell degeneration (Kovács et al., 2021).

Caffeine showed immuno‐protective effects in laboratory (in vivo and in vitro) experiments (Kovács et al., 2021). Reduction of T‐cell proliferation and impairment in the production of Th1 (IL‐2 and interferon‐gamma), Th2 (IL‐4, IL‐5), and Th3 (IL‐10) cytokines from human blood were observed due to the effect of caffeine which is indicative of its function to suppress the human lymphocytic activity. Caffeine may decrease endotoxins' lipopolysaccharide (LPS)‐induced inflammatory reactions according to in vitro findings (Hwang et al., 2016). A recent human investigation in obese females found that caffeine suppressed LPS (Silva‐Ramos et al., 2022). The study also discovered that caffeine reduces obesity‐related metabolic side effects such as increased LPS, insulin action, glucose homeostasis, and testosterone levels after a high‐intensity exercise lifestyle intervention. These studies show that when caffeine is paired with other lifestyle factors, such as exercise, the metabolic, and immunoprotective advantages are enhanced.

### 2.3 Spices

#### 2.3.1 Garlic

**FIGURE 4:**
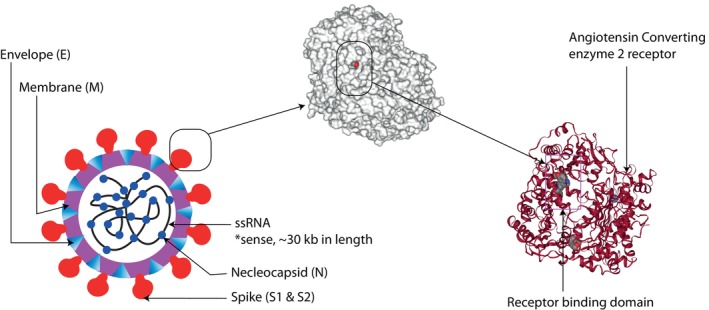
Schematic depiction of the primary SARS defense mechanisms. Adapted from Attah et al. (2021).

Garlic (*Allium sativum* L.) possess a powerful antiviral agent whose every day consumption can be a great way to improve one's immune system (Rouf et al., 2020). It is found to have high levels of organosulfur compounds like quercetin and allicin which are associated with inhibition of viral infection (Wu et al., 2016). Especially allicin can be credited as the attributing factor for the health‐benefiting properties of garlic. These organosulfur substances can hinder virus adhesion to host cells, alter viral genome transcription, and translation in host cells, and interfere with viral assembly. Several studies have shown that garlic can be effective against influenza A and B (Ahmed et al., 2017), HIV, HSV‐1 (Patel et al., 2004), and viral pneumonia, rhinovirus (Chen et al., 2011). As a result of its bioactive components and antiviral mechanism, allicin from garlic could be regarded as an excellent choice for preventing the severity of viral infection, particularly SARS‐CoV‐2‐related RNA viruses (Mösbauer et al., 2021). In Figure 4, the defense mechanism of natural metabolites against SARS‐CoV‐2 has presented.

#### 2.3.2 Star anise

Star anise (*Illicium verum*) is a prominent medicinal plant found throughout the southern Asian continent (Patra et al., 2020). The flower‐shaped spice includes shikimic acid, which is utilized as a basis material in the manufacture of Tamiflu, an antiviral drug. Boiled star anise added to drinks such as green tea or black tea can be quite effective as an antiviral medicine (Patra et al., 2020). Linalool, Anethole, Quercetin, Gallic acid, Shikimic acid, and Limonene are some of the key health‐promoting components present in star anise. These chemicals may work together to provide star anise its antioxidant, anti‐inflammatory, and antibacterial benefits (Patra et al., 2020). The use of star anise as an alternative to antiviral medicine has been the concept of several studies like Alhajj et al. (2020), which have produced promising results. The preliminary findings of this study imply that star anise might be a natural alternative to antiviral medicines (Alhajj et al., 2020). Infections and inflammation of the respiratory tract, swine flu, gastrointestinal disturbance, loss of appetite, and flatulence, can be helped with star anise as a viable treatment component to a certain extent.

#### 2.3.3 Ginger

Ginger (*Zingiber officinale*) and its aqueous extracts might be useful in preventing foodborne virus contamination, especially for those foods that are commonly not processed sufficiently before consumption (Abd El‐Wahab et al., 2009). Fresh ginger displayed antiviral efficacy against HRSV in both HEp2 (Human Epidermoid Carcinoma) and A549 (adenocarcinomic human alveolar basal epithelial cells) cells, according to a study conducted by Chang et al. (2013). Furthermore, aquatic extract of fresh rhizome of ginger showed outstanding activity against the highly contagious Chikungunya virus which otherwise has very little antiviral treatment available (Kaushik et al., 2020). Additionally, ginger contains zinc, magnesium, and chromium, which help blood circulation and protection against fever, chills, and excessive sweating, improve the absorption of essential nutrients for the body, help digestion, and reduce pain and inflammation (Shahrajabian et al., 2019).

#### 2.3.4 Turmeric

Turmeric (*Curcuma longa*) contains a huge number of bioactive compounds filled with medicinal qualities. It contains active elements like curcumin a phytochemical that possesses strong antioxidant, anti‐inflammatory, and antiviral activities. Regular intake of turmeric milk can trigger immunity response and help combat viral infections (Kundu et al., 2021). In a study, curcumin supplementation was provided to a group of people who received their first Covid‐19 vaccination, until 4 weeks after the second vaccination. The average antibody formed in that group of people showed significant increased immunity than the control group (Widjaja et al., 2022). Curcumin had the potential as a complementary supplementation during the period of Covid‐19 vaccination as it increased antibodies produced post vaccinations (Abdelazeem et al., 2022).

#### 2.3.5 Black cumin

Black cumin (*Nigella sativa*) is a nutritious herbaceous plant that has been utilized in the treatment of respiratory diseases, diabetes, hypertension, inflammation, and influenza since ancient times (Khader & Eckl, 2014). The presence of high levels of Thymoquinone (TQ) has been found in black cumin which can boost the humoral immune system and induce the expression of cytokines resulting in early viral clearance. Similar studies have also shown significant antiviral efficacy against the avian influenza virus, murine cytomegalovirus (Salem & Hossain, 2000), and hepatitis C virus. TQ analogs (chloroquine and hydroxychloroquine) have recently been reported to be efficient therapeutic medications for treating SARS‐Cov‐2 (Barakat et al., 2013; Kulyar et al., 2021).

#### 2.3.6 Cinnamon

Cinnamon (*Cinnamomum zeylanicum*) is a common culinary ingredient that has been quite useful as a home remedy for millennia in different countries. Cinnamaldehyde, cinnamyl alcohol, coumarin, cinnamic acid, and eugenol are the main components of cinnamon bark (Hong et al., 2012). Some of its health benefits have been established in studies like (Shen et al., 2012) and Sillapachaiyaporn et al. (2021) which conclude defense against viral infection, efficiency in preventing HIV‐1 and HIV‐2, stopping viral DNA replication by inhibiting HIV protease.

Cinnamaldehyde inhibited the highly pathogenic H7N3 influenza A virus both in vitro and in vivo (Munazza et al., 2016), and the HSV‐1 virus. Coumarin substances decreased the expression of several genes limiting viral replication by inhibiting the production of viral polymerase (Mishra et al., 2020). Cinammon's antiviral efficacy was also quite apparent against the viral diseases like Dengue and Chikungunya, with 4‐thiazolidinone‐coumarin derivatives possibly limiting viral protease activity. Furthermore, some derivatives of coumarin such as 7‐[6‐(2‐methylimidazole) hexyloxy] coumarin, displayed antiviral efficacy by preventing virus entry into the host cell by destroying glycoprotein (Chen et al., 2018). As a result, the active ingredients of cinnamon could be used as therapeutic antiviral agents, particularly against SARS‐CoV‐2, because they share a common mechanism against RNA viruses.

#### 2.3.7 Licorice root

Licorice root (*Glycyrrhiza glabra*), has been utilized as a traditional cure in various cultures from ancient times. Licorice root is regarded as a life‐enhancing substance, and several researches has revealed that it has a wide range of pharmacological properties (Matsumoto et al., 2013). Glycyrrhizin (GL) and 18‐glycyrrhetinic acid (GA) are two of the components of licorice root that have antiviral activities. Previous research showed that GL has substantial antiviral activities against SARS‐CoV‐2‐related viruses, which is why it was considered a treatment option for treating COVID‐19 during the pandemic. According to Matsumoto et al. (2013), licorice root can aid in the treatment of chronic hepatitis C. Also, existing literature evidenced that GL therapy helps prevent HIV‐related viral infection. According to Wang et al. (2015), GL contained in the licorice root can be an antiviral medication for the treatment of influenza as it inhibits influenza virus polymerase activity. GL is also suggested to have sufficient immune‐stimulant properties by increased T lymphocyte proliferation against duck hepatitis virus (DHV) (Soufy et al., 2012). GL‐resistant infections included enterovirus 71 (EV71), coxsackievirus A16 (CVA16), and HSV type 1 (HSV1) (Wang et al., 2015). When seen as a whole, the antiviral potentiality of the licorice root can contribute dramatically towards the remediation of potential viral infections.

#### 2.3.8 Black pepper

Black pepper (*Piper nigrum* L.) includes a large number of bioactive chemicals that have been demonstrated to be beneficial to human health, including antiviral, anti‐inflammatory, antipyretic, and immunological effects. The principal phytochemicals found in peppers are alkaloids, terpenes, flavonoids, steroids, etc. (Ali et al., 2018).

**FIGURE 5:**
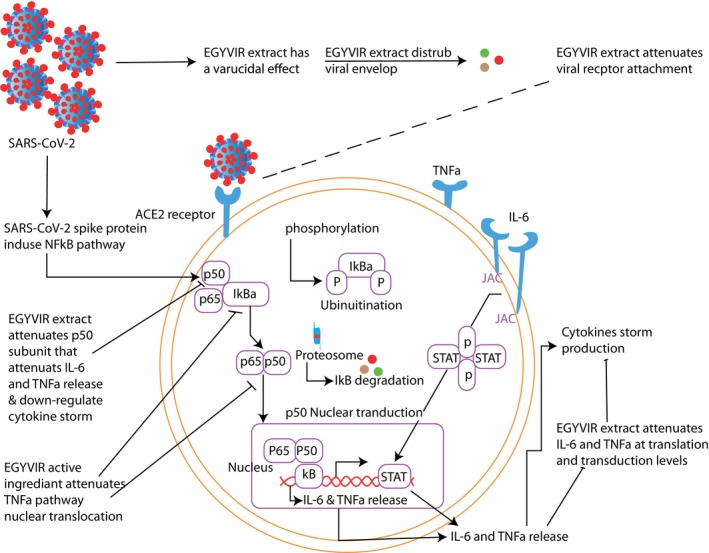
EGYVIR molecular mechanism as a virucidal and cytokine storm disturbance via NF‐Kb pathway. Adapted from Roshdy et al. (2020).

Piperine, an alkaloid, which helps black pepper exhibit potential antiviral action against coxsackie virus type B3 (CVB3), vesicular stomatitis Indiana virus (an enteric virus), and human parainfluenza virus (a respiratory infection causing virus) (Taylor et al., 2020). Black pepper can also increase the number and efficiency of white blood cells, assisting the body in developing a strong defense against invading germs. Piperine also inhibited proliferative responses induced by lipopolysaccharide (LPS) and immunoglobulin α‐IgM antibodies (Bernardo et al., 2015). In Figure 5, the molecular mechanism of an herbal extract, namely EGYVIR, formed by mixing a certain proportion of black pepper and curcumin in a controlled environment, has been depicted schematically. The antiviral activity of black pepper in the fight against SARS‐CoV‐2 can be explained in two stages, the inhibition of the nuclear translocation of NF‐kβ p50 and the exhibition of an in‐vitro virucidal effect against the infecting virus (Roshdy et al., 2020).

#### 2.3.9 Drumstick tree

Drumstick tree *(Moringa oleifera* L.) is a medicinal and nutritious plant with a wide range of applications. All components of the tree i.e., bark, root, leaf, seeds, pod, gum, fruit, and flowers are rich in vitamins, antioxidants, minerals, proteins, nitrile glycosides, and phenolics, but the leaves are the most nourishing (Anwar et al., 2007). It has a unique combination of bioactive components such as zeatin, beta‐sitosterol, quercetin, kaempferol, caffeoylquinic acid, etc. (Modi et al., 2010). Antiviral, antibacterial, anti‐diabetic, antipyretic, anti‐inflammatory, antihypertensive, antioxidant, and antifungal actions have been documented in various components of this plant (Singh & Navneet, 2018). The presence of saponins, alkaloids, glycosides, terpenoids, and phenolic chemicals, in addition to its diverse range of medical uses, is responsible for the wide antiviral activity.

Previous studies such as Shaji et al. (2021) indicated that thiocarbamate of drumstick tree was particularly effective at inhibiting the activation of the tumor‐promoting viral Epstein Barr virus (EBV). The leaf of *M. oleifera* could be used as a preventative or therapeutic treatment for HSV‐1 infection (Lipipun et al., 2003). According to literature, the fruit extract of drumstick tree along with the plant's seed exhibited considerable anti‐hepatitis B virus (HBV) activity, and was also quite effective against Newcastle Disease Virus (NDV) and Infectious Bursal Disease (IBD) virus (Aslam et al., 2016; Rahman et al., 2021).

### 2.4 Fruits and vegetables

Regular consumption of plant foods such as whole grains, fruits and vegetables, and other items has been associated with a reduced risk of the advancement of chronic diseases. Fruits, vegetables, pulses and legumes, and other plant foods contain a wide range of nutrients and bioactive compounds, including phytochemicals, vitamins, minerals, and fiber (Liu, 2013).

Citrus fruit has antiviral activity. Arena viruses that cause viral hemorrhagic fever are blocked from entering cells by tangerine, a citrus peel extract (Tang et al., 2018). Apple beverages have antiviral effects. Investigations on the antiviral properties of a variety of apple drinks were conducted against poliovirus 1 and coxsackievirus B5. Freshly made apple juice was found to be highly antiviral, but its activity was less resistant to heat and storage than commercial juice. Following ultrafiltration, antiviral activity was observed in fractions with molecular weights greater and lower than 10,000, showing that the active ingredient was present in both pulp and skin. In virus‐apple juice complexes, gelatin, Tween 80, serum, or polyethylene glycol failed to reactivate viral infectivity (Konowalchuk & Speirs, 2006). Apple cider vinegar (ACV) is an example of a widely used yet scientifically unproven treatment. Findings show that ACV has unmistakable antibacterial action at maximum power doses (Gopal et al., 2019).

Mushroom is another example of antiviral food. Polysaccharides, organic acids, steroids, lipids, and tetracyclic triterpenes are just a few of the substances found in mushrooms that have antiviral properties. Those compounds also impede the formation of viral nucleic acids, viral enzymes, and viral adsorption into human cells. *Lentinus edodes* (shiitake), and polypore mushrooms efficiently fought off the flu‐causing influenza viruses, according to Lipipun et al. (2003). Both *Daedaleopsis confragosa* and *Ischnoderma bezoinum* species exhibited Influenza A virus‐neutralizing properties (Teplyakova et al., 2012).

By restricting virus adsorption onto cells, inhibiting viral nucleotide synthesis enzymes, and boosting cellular immunity, several mushrooms, including *Ganoderma* spp. and *Piptoporus* spp., have demonstrated antiviral efficacy against the chickenpox and HIV viruses (Ghosh et al., 2022). Additionally, by blocking HIV‐1 reverse transcriptase, velutin from *Flammulina velutipes* work as antiviral food. In addition to their antiviral capabilities, many mushrooms also have immune‐modulatory attributes, which helps researchers to develop immune‐modulating drugs from mushrooms. For example, both Himematsutake and Agaricus mushrooms contain FIo‐a‐β, FA‐2‐b‐Md immune modulators, while Shiitake and Golden Oak mushrooms contain KS‐2, Lentinan, and LEM immune stimulators. SCG and GLP(AI), ganoderans, ganopoly, and protein LZ 8 are immune modulators found in cauliflower mushrooms and Reishi, and Lingzhi mushrooms, respectively.

### 2.5 Functional food

Functional foods are now generally recognized for the prevention and treatment of major non‐communicable diseases (NCDs), particularly those characterized by inflammatory and oxidative stress‐related disorders like diabetes and cardiovascular disease. A variety of oily fish, fruits, vegetables, nuts, olive oil, and legumes are all regarded as functional foods since they include natural levels of nutraceuticals such as terpenoids, polyphenols, flavonoids, sterols, pigments, alkaloids, and unsaturated fatty acids. It has been suggested that bioactive peptides, which are inherently present in fruits and vegetables are developed as nutraceuticals according to their amino acid chain length, molecular weight, or peptide composition, which can cause a broad spectrum of physiological responses linked to the gastrointestinal, cardiovascular, neurological, and other hormonal functions of the human body (Zaky et al., 2021).

Adequate dietary intake, as well as supplementation with such functional foods, helps to keep the human body at optimal levels, which benefits numerous components of the immune system and helps to avoid viral infections. A balanced diet and the addition of these functional items to the diet assist to maintain ideal body conditions, which supports several immunological factors and also helps prevent CDs.

In order to simultaneously tackle NCDs and CDs in high‐risk groups, it is particularly important to look into the impact of functional foods on CDs, specifically COVID‐19. Age, obesity, heart disease, hypertension, and diabetes have all been associated with greater COVID‐19 infection and mortality rates (Escobedo‐de la Peña et al., 2021). According to COVID‐19 statistics in England, for instance, over a third of COVID‐19‐related death was associated with type‐2 diabetes, and 86% of obese COVID‐19 infected people needed ventilatory support than healthy‐weight infected people (47%) (Robinson et al., 2021). Nowadays, diabetes is regarded as a risk factor for the development and prognosis of COVID‐19 (Guo et al., 2020). A topic of rising importance is the incidence of NCDs, especially diabetes among high‐risk groups. In order to avoid NCDs and CD multimorbidity, the best “immune‐enhancing” functional foods should be combined with behavioral lifestyle measures, including exercise.

Many dietary programs comprise functional foods that have previously been advised for NCD prevention, especially the vegetarian diet, the Mediterranean diet (MD), the Nordic diet, and its integration with other lifestyle strategies. All necessary nutraceuticals with disease‐preventive, anti‐inflammatory and antioxidant properties may be found in fermented foods, dairy products, teas, tree nuts, and olive oil. Olive oil contains monounsaturated fatty acids (MUFAs), such as oleic acid. Tree nuts like walnuts contain polyunsaturated fatty acids (PUFAs), notably alpha‐linolenic acid. Docosahexaenoic and eicosapentaenoic acids are present in fish oil. Fruits and vegetables contain significant levels of flavonoids, polyphenols, and antioxidants. The prevalence of such functional foods and their constituent parts vary by region, but their cardiometabolic preventive effects in lowering key NCDs and fatality risks are widespread. By enhancing and protecting the immune system and having antiviral defensive actions, the challenge is to transform these functional properties into the protection of new CDs like COVID‐19 (Eng et al., 2019).

Resveratrol, a naturally occurring polyphenol, is a strong antioxidant that may be found in foods including grapes, mulberries, and peanuts. Peanuts, pistachios, grapes, red wine, cranberries, white wine, strawberries, blueberries, and even cocoa and dark chocolate are high in resveratrol, which can help in combating fungal infection, UV radiation, stress, and damage. It has antiviral effects against several viral infections (in vitro and in vivo) as well. Trans‐ and cis‐isomeric versions of resveratrol are available. Resveratrol was shown to significantly limit MERS‐CoV replication in vitro by inhibiting RNA synthesis, as well as having additional pleiotropic effects. Studies have shown that resveratrol and indomethacin are useful supplements for SARS‐CoV‐2 (Barh et al., 2020). For the consistent and repeatable synthesis of resveratrol and its derivatives, researchers used hairy root lines from the peanut plant *Arachis hypogaea* (Medina‐Bolivar et al., 2007).

Amla, citrus fruits, red peppers, and yellow peppers are some of the foods that are high in vitamin C content which boosts the immune system in the human body (Colunga Biancatelli et al., 2020). Coconut oil possesses lauric and capric acids. Cold‐pressed raw coconut oil are crucial for boosting the immune system to combat infections (Angeles‐Agdeppa et al., 2021).

### 2.6 Honey

The body's defenses are strengthened by honey, a natural immune booster. Along with organic acids like gluconic acid, honey possesses the amino acid proline, which has several beneficial effects. According to Hossain et al. (2020), using honey as a treatment to stop the varicella‐zoster virus's zoster rash is a successful therapeutic method.

Methyl glyoxal, a component of honey, possesses antiviral characteristics that are effective against influenza and respiratory syncytial viruses (Wong et al., 2021). Minerals such as magnesium, potassium, sodium, calcium, phosphorus, zinc, iron, cobalt, and copper are all present in honey. However, multiple investigations have found that potassium is the most prevalent mineral in diverse types of honey. For the treatment of SARS‐CoV‐2‐infected patients, potassium ion supplementation in honey demonstrated a higher likelihood of recovery (Abedi et al., 2021).

## 3 OPPORTUNITIES AND CHALLENGES

Functional plant food possesses the ability to enhance not only the immune system and treat illness but also the overall health. Plant‐based antiviral agents may be able to control the release of proinflammatory cytokines, inhibit viral development in host cells, and change the RAA system's molecular pathways. Since food includes multiple ingredients, the antiviral effects might be a result of synergistic effects through multiple mechanisms.

It is necessary for the physician to proceed with caution while administering these antiviral agents even if the person is in good health, since there has been a lot of contradicting information concerning these agents. These may be connected to the development of known or unknown harmful side effects. Furthermore, very few preclinical or clinical studies of these antiviral agents have been carried out, suggesting that full phase study is required (Nugraha et al., 2020).

Functional food can prevent and cure disease, and the more we understand about it, the better equipped we are to create a diet that will help us achieve our health goals. The issue is that we must choose the correct foods, those that will raise us while avoiding the low‐quality foods that will hamper the normal metabolic and physiological processes (Lange, 2021). The risk of diseases has been linked to dietary patterns. While certain foods can make chronic conditions worse, others have tremendous therapeutic and preventative benefits. As a result, many people believe that eating may cure illnesses. However, in some circumstances, diet alone cannot and should not be used to substitute medicine. Many diseases may be avoided, treated, or even cured by altering one's diet and lifestyle, while others cannot (Hanna et al., 2021).

Cytotoxic evaluation is an essential aspect of research concerning the development of novel and effective antiviral medications. Functional compounds with robust antiviral activity must be proven to be free of toxicity and cytotoxicity in an appropriate cell culture system. Different active components of plants must be extracted using appropriate solvents for testing (Hanna et al., 2021).

## 4 CONCLUSION

Viral infection is a global public health issue that affects a vast number of people. During the last decade, there has been a considerable increase in the number of instances of viral infections involving people of all ages. For a long time, plants have been utilized to treat viral infections. Plants are therapeutically beneficial in viral diseases; nonetheless, declaring that all of these plants may be blindly recommended to infected people would be unjustified. Following the pharmacological validation of plant based antiviral agents for human consumption, all evaluation characteristics such as inter compounds interaction, as well as hazardous consequences must be established and validated. The synergistic effect of antiviral foods and medication on immune boosting is another focus area. Since, human defense mechanism is greatly affected during viral infection, eating antioxidant and antiviral agent‐rich foods along with conventional medication would be more effective.

## AUTHOR CONTRIBUTIONS

**Md. Shofiul Azam:** Software (supporting); visualization (equal); writing – original draft (lead); writing – review and editing (equal). **Md. Nahidul Islam:** Conceptualization (lead); project administration (lead); writing – original draft (lead); writing – review and editing (equal). **Md. Wahiduzzaman:** Visualization (lead); writing – review and editing (supporting). **Mahabub Alam:** Writing – review and editing (equal). **Akib Atique Khan Dhrubo:** Writing – review and editing (equal).

## CONFLICT OF INTEREST STATEMENT

The authors declare no conflict of interest.

## ETHICAL APPROVAL

No ethical approval was required for this article since no human or animal study was Conducted.

## Data Availability

Data for this research were online publications from the Web of Science as contained in the reference section.
